# Challenges to belief systems in the context of climate change adaptation

**DOI:** 10.4102/jamba.v10i1.508

**Published:** 2018-09-06

**Authors:** Brechtje S. Jooste, Jon-Vegard Dokken, Dewald van Niekerk, Ruth A. Loubser

**Affiliations:** 1 Unit for Environmental Sciences and Management, North-West University, South Africa; 2 Department of Sociology and Human Geography, University of Oslo, Norway; 3 School of Philosophy, North-West University, South Africa

## Abstract

This article focuses on the social aspects of climate change and explores the interrelationship between belief systems and adaptation. The links and interaction between external and internal realities are examined from the perspective of contextual vulnerability, with a focus on the multifaceted structure of belief systems. The aim was to determine those challenges regarding climate change adaptation that are caused by a community’s belief system and to make recommendations to overcome them. Diverse perceptions of climate change and beliefs from three townships in the North-West Province of South Africa were collected and analysed using Q-methodology, finding five distinct worldview narratives. These narratives were named naturalist collectivist, religious, religious determinist, activist collectivist and structural thinker. It is recommended that policymakers aim to address diverse views and should be informed by factors that increase resistance to belief revision. Information should be framed in ways that foster the perception of internal control, are clearly evidence based and encourage a desire to learn more.

## Introduction

As people’s realities often reside within the walls of their own experience, adaptation to external stimuli is buffered. Perception is coloured by filters such as beliefs, and therefore in order to study responses to environmental change, the study of internal processes is very helpful. Discourses on belief systems and climate change adaptation are prompted by the discrepancy between scientific data and public perception on climate change (Antilla 2005; Boykoff & Boykoff 2004, 2007a, 2007b; Ohwo 2015; Poortinga et al. 2011; Weingart, Engels & Pansegrau 2000).

Climate change is very much a physical reality, with scientific measurements taken of a myriad subcomponents of the phenomenon, but the mental construct of climate change is not the physical reality. The construct ‘climate change’ as a social phenomenon can be framed according to diverse ideological perspectives (Hulme 2011:xxvi). Transdisciplinary, holistic research has been encouraged to better understand the anthropological bearing of climate change (McNeeley & Lazrus 2014:509; Pendergraft 1998:645; Schipper 2007:9;). Hernes (2012:89) argues that climate change is but one of a convergence of crises that include political, social and environmental dilemmas and that these dilemmas should be looked at simultaneously to avoid an impending disaster. The eco revolution accompanying the climate movement can, for example, also be viewed as a peace movement to prevent resource wars, recognising our ‘addiction’, or conditioning, to fossil fuels. Climate change is also intertwined with urgent short-term concerns such as water shortages and sharply rising food prices (Kings & Wild 2015).

Looking at climate change in this way illuminates the connections between climate change adaptation and belief systems. One component of adaptation is the changing of beliefs and the reorganisation of belief systems. The pressure that climate change and belief systems exert on each other is noteworthy in this regard; however, there are more linkages. For example, regulating adaptive policy in such a manner as to take context-specific perceptions (shaped by belief systems) into account. Clearly, belief systems and climate change adaptation are interrelated. Laying these interrelations bare can provide valuable guidance for the adaptation process, and therefore, it is fitting to investigate these two concepts more closely.

### The structure and function of belief systems

Climate change presents humanity with a multi-factored challenge; therefore, internal subjective components of crises, such as beliefs, should be studied in concert with objective research and active engagement in climate change (O’Brien & Hochachka 2010:3–4). This is likely to determine the buy-in of an idea. It is also true that beliefs can only be addressed effectively when they are understood well (O’Brien & Wolf 2010:237). In order to understand specific beliefs, it is necessary to first study what beliefs are, how they work, and under which circumstances they change.

A belief has three components that, if held consciously, a person strives to keep more or less in harmony: the cognitive, the affective and the behavioural (Rokeach 1968:113–114). Not all beliefs have equal impact on human beings, and different authors have visualised networks of beliefs in different ways. This architecture of beliefs offers explanations of how and why changes in beliefs occur and also why, in the face of overwhelming evidence, they sometimes don’t.

Wolterstorff (1976:11–16) divides beliefs into three categories based on their origin and function. Lying at the centre of a belief system and regulating the organisation of more peripheral beliefs, control beliefs represent a person’s authentic commitment. Data background beliefs are those beliefs held about devising theory and data beliefs are the theory itself. Loubser (2013:13) looks at the organisation of beliefs and distinguishes between pre-theoretical, theoretical and combination structures. An example of a structure that is both theoretical and pre-theoretical is a paradigm. According to Rokeach (1968:124), pre-theoretical structures can be held either consciously or subconsciously. Control beliefs and data background beliefs both fall into this category, whereas data beliefs are theoretical (and conscious).

Not all beliefs are equally important (Wyer & Albarracín 2005:276). The most central and therefore connected beliefs are axiomatic and derive directly from object encounters; these are also the minimum guarantee for stability and include basic beliefs such as person, object and self-constancy (Rokeach 1968:7). Connectedness and importance or centrality of beliefs are correlated, although both intensity and verifiability stand independent of centrality (Rokeach 1968:5,13). Shared beliefs (beliefs held by many others as well) and existential beliefs are central, and so are beliefs that spring from direct experience (Rokeach 1968:6). Non-primitive beliefs are distinguished from primitive ones as those that differentiate as one matures to round out one’s world picture ‘realistically and rationally to the extent possible, defensively and irrationally to the extent necessary’ (Rokeach 1968:9). Rokeach also identifies authority beliefs, which are beliefs about positive referents that a person trusts to have the correct information. These beliefs are determined by social structure, education and experiences, and also include negative referents, which are seen as having incorrect information.

Knowledge beliefs are organised via concepts into conceptual systems (Thagard 1992:20–21). According to Hernes (2012:113) if one belief changes, it can cause a rearrangement of the whole network, because constructs of causal relations are logically linked, whereas Rokeach (1968:2–3) warns that these connections are not necessarily logical. Inconsistencies sometimes only become apparent with the realisation that a new belief causes a logical contradiction, or when there is no specific compartment for a new belief (Rokeach 1968:117–118). In this case, either the belief, view or commitment will be revised (Wolterstorff 1976:88). Unnoticed inconsistencies within a belief system can be explained by either compartmentalisation or ‘an uncritical internalization of contradictory values and attitudes’ (Rokeach 1968:167–168). Consistency is most important within our self-esteem framework and only then according to logic and reality (Rokeach 1968:164).

Beliefs are created for a purpose and therefore have motivational roots (Wyer & Albarracín 2005:306). If an emotional charge accompanies a belief, it will be used often and more easily (Marsh & Wallace 2005:11). We tend to follow the path of least resistance in these very dynamic frameworks, and an emotion-induced rejection of best explanation (Thagard & Findlay 2010:342) can be this easier route. One is also prone to infer certain propositions according to a completion principle (Wyer & Albarracín 2005:287), as uncertainty is unfavourable in a coherent framework.

If new information causes the coherence of the belief system to increase, you will be likely to change a pre-existing belief (Thagard & Findlay 2010:333). Changes usually entail content, but it can also refer to an organisational change only. An example of this would be a shift from a literal to a figurative perspective (Rokeach 1968:135). Thagard and Findlay (2010:336) refer to a Gestalt switch or a tipping point for change. With climate change, it is sometimes a slow process. Weather patterns are observed casually over time and gradually, but with a specific moment of realisation, change is recognised (Hernes 2012:114).

Beliefs are built on values according to Thagard and Findlay (2010:331), who explain global warming critics’ trust in the free market, property rights and technology to encourage environmental responsibility and solve global warming on their underlying values of liberty, economic stability and technology. Values are cognitive constructs that lie at the core of our conscious beliefs (O’Brien & Wolf 2010:3) and are decisive factors in designating causality (Rokeach 1979:2). Values are abstract and idealistic. A hierarchical organisation of values is learned and acts as a guide in conflicts between different end-state existences or modes of behaviour (Rokeach 1968:124,161; 1979:2). They form a unifying structure for beliefs by maintaining attitudes, guiding actions and moral judgements, and justifying oneself and others’ behaviour throughout different situations (Rokeach 1968:160). It is a prejudgement and sometimes emotionally charged, which explains why evidence might not be sufficient for change (Rokeach 1968:131). In these terms, values can be equated to Wolterstorff’s authentic commitment: a person’s authentic commitment or control beliefs are the guide by which all new information is structured and it gives meaning to information. A worldview can be said to have the same function, as it categorises the world and relationships in it (Loubser 2013:16).

The reason that a value is such a dynamic concept is because of its motivational element (in addition to the cognitive, affective and behavioural elements that a belief and an attitude also share) (Rokeach 1968:158). Attitudes are intertwined subsystems that operate as a relatively stable framework to organise underlying beliefs around an event, a context or an object, with the function of helping one manage by easing one’s decisions (similar to values, it can act as prejudgement). In this way, coherent cognitive frameworks develop (Marsh & Wallace 2005:8). An attitude becomes more prevalent when more values anchor it and determines the nature and scope of behaviour accordingly.

Kuhn (1962:122) noted that one’s perception and interpretation are biased in favour of one’s current beliefs,^1^ and Lorenzoni and Hulme (2009:383) stated that interaction with an object or situation is largely determined by beliefs about it. Regarding climate change, the fundamental view one has about nature and society, including social interactions, help determine one’s risk perception of it (McNeeley & Lazrus 2014:506). Foundational beliefs about the relationship between God, humankind and nature influence both perceptions of climate change and the categories for solutions, because it affects who we blame. It affects how we value ecosystems, nature and human dignity and how we judge humanity’s purpose and destiny (Hulme 2011:144–146).

The relevance of perception to adaptation extends to every aspect of its definition, including risk perception, perception of vulnerability and perception of capacity. Perception is an important link between beliefs and adaptation.

### Climate change adaptation

Climate change adaptation in social systems is defined by the Intergovernmental Panel on Climate Change (IPCC) (2012:5) as ‘the process of adjustment to actual or expected climate and its effects, in order to moderate harm or exploit beneficial opportunities’. Eriksen et al. (2011:2) define climate change adaptation as reducing climate’s adverse effects on well-being and health, while seizing opportunities from the climatic environment. The amelioration of negative consequences together with the utilisation of opportunities seems to saturate most definitions of adaptation. Adjustment includes systemic, behavioural, as well as internal cultural and personal changes (O’Brien & Hochachka 2010:2). Change can be threatening, and unless it is internally accommodated and prepared for, it may very well be resisted (Ensor & Berger 2009:33).

Adaptation can be viewed from two perspectives that should ideally line up in the end and complement each other. Autonomous adaptation is the natural process of responding to change from within a community. On the other end of the spectrum is purposeful adaptation, which is policy, regulations and other ways of control external to the community. There is a very important flow of information between the community and the government in this regard. An upwards flow contributes to a local map of reality and a downwards flow conveys relevant scientific discoveries (Ensor & Berger 2009:21–22). For the public to understand new knowledge and integrate it into their framework of reality, science needs to be interpreted and communicated. In this way, a shared account is created that leads to a shift in mentality, which will be strengthened into a unified narrative if different accounts converge over time (Hernes 2012:96–116).

Knowledge is an important part of adaptive capacity, which is studied as part of the process of adaptation (in other words autonomous adaptation). The capacity to adapt encompasses many different abilities, resources, processes, practices and systems, and is countered by vulnerability. Vulnerability and adaptation are inversely related, and therefore, vulnerability is an essential latent component of adaptation (Schipper 2007:3). The way in which vulnerability is defined influences the way that adaptation is perceived within the context of study. For this reason, the use of vulnerability within this study is delineated below.

The United Nation’s International Strategy for Disaster Reduction (UNISDR) defines vulnerability as ‘conditions determined by physical, social, economic and environmental factors or processes, which increase the susceptibility of a community to the impact of hazards’ (Birkman 2006:12). Psychological factors are arguably included under social factors, but intentionality is missing from this definition (Wisner 2015). Wisner et al. (2004:11) propose ‘characteristics of a person or group and their situation that influence their capacity to anticipate, cope with, resist and recover from the impact of a natural hazard’. This definition shifts the focus to non-physical attributes. O’Brien and Wolf (2010:232) stress that vulnerability is determined in large part by how changes are appraised. Birkmann (2013:39) puts vulnerability as internal risk factor at the core of a widening concept of vulnerability, increasing in scale and complexity. At the core, it is strategically positioned to cause a ripple effect. Of course, both internal and external aspects are relevant, but it does appear that internal aspects are neglected in official definitions.

Kelly and Adger (2000:327), O’Brien et al. (2004:2) and Ensor and Berger (2009:14) recommend using a contextual definition of vulnerability, viewing vulnerability from a starting-point approach, rather than from a possible outcome. It is a definition that takes into account a community’s current situation and aims to understand established practices within a community, taking into consideration the interaction of social, political, economic and environmental processes. In this study, vulnerability is considered contextually with an emphasis on intrinsic vulnerability. With this definition in mind, adaptive capacity can now be considered in more detail.

According to the IPCC, adaptive capacity is ‘the ability of systems, institutions, humans, and other organisms to adjust to potential damage, to take advantage of opportunities, or to respond to consequences’ (IPCC 2014:1758). Importantly, adjustment includes overcoming challenges to standing beliefs and worldviews (O’Brien & Hochachka 2010:90).

The International Union for Conservation of Nature (IUCN) created a list of characteristics for individual and community adaptive capacity. On the individual level, this includes risk perception, coping ability, interest to change oneself, capability to reorganise, learn and plan, environmental knowledge and awareness (including beliefs and attitudes), equity perception in resource access, attachment to place and occupation, formal and informal networks, family features, employability, financial situation (including access to credit and diversity of income) and access to information about climate change (including skills development and technology) (Marshall et al. 2009:12–15). On a community level, adaptive capacity is made up of the ability to learn, experiment and reorganise, community assets (including social and human capital), flexibility, gender relations, social norms and environmental institutions, the presence of corruption and markets (Marshall et al. 2009:16–18).

Availability of knowledge is an important aspect that has already been touched upon and that has to be addressed from a policy level. Willingness to learn depends on the flexibility of a belief system. Functional and structural aspect of individual and communal belief systems are indeed intertwined with many aspects of adaptive capacity. For the purposes of this study, risk perception, social capital and indigenous knowledge will be considered in more detail.

Natural adaptive responses have developed in the form of indigenous resource management (Berkes 1999:159), but as environmental changes become more pronounced, humankind’s relationship with their environs evolve (O’Brien & Wolf 2010:233–234,237). Accumulated indigenous knowledge is fast becoming obsolete, or at the very least vastly insufficient, in this fast changing landscape. Paired with an ever increasing population growth, additional problems such as loss of biodiversity only confirm the bleakness of the situation (Ensor & Berger 2009:3).

Although climate change is an abstract concept, manifestations such as erratic weather patterns and frequent record high temperatures (Kings 2015) are categorically observed all over the world (Turner & Clifton 2009; Piccolella 2013). Flexibility of human systems such as social networks, livelihoods and cultural traditions are instrumental in mitigating impact and promoting successful adaptation (Schipper 2007:6). In traditional worldviews, cultural identity is often important. From a policy level perspective, the local assessment of power dynamics and value in decision-making is especially important when considering new adaptation strategies (Adger, Lorenzoni & O’Brien 2009:315, 321).

Danger can be defined internally from a bottom-up approach and is then made real by the people’s perception, in contrast to an external scientific valuation (Hulme 2011:193). In this manner, risk perception happens within a social network. If it differs from the external valuation, a gap in communication can be identified. Perception of capacity is pivotal for action. Wolf (2011:25) warns about inaction when either the perception of individual agency or controllability is low.

Adger (2003:392), focusing on the community asset, social capital, divides it into bonding capital, pointing to kinship and camaraderie, and networking capital. It is this latter type that is of special importance in adaptation and it points to relationships based on shared interests, without necessarily shared backgrounds. Mutuality and commitment builds up relationships that act as channels through which material and non-material resources are exchanged. In terms of bonding capital, to change one’s beliefs may sometimes mean investing in a new intercommunity and identity (Hernes 2012:116). People also react to each other’s responses leading to a possible ‘collective conversion’. An event can, for example, cause a broader awareness of an issue and lead to the change of diverse attitudes from associated areas (Hernes 2012).

Within contextual vulnerability, adaptation means taking measures against the causes of vulnerability surrounding the anticipated disaster and negative impact on livelihoods (Ensor & Berger 2009:16). Vulnerability is engendered by a variety of processes and factors within a social or ecological system (O’Brien et al. 2007:75). Justice and equity should be paramount in the process of empowering people to respond better to change by altering their situation or surroundings (O’Brien et al. 2007:76).

Within developing countries, added pressure from climate change can stall development by shifting the focus from sustainable development to adaptation. Schipper (2007:10) advocates for an adaptation to climate change that is ‘a paradigm for development, where adaptation is fostered by a process of sustainable development and vulnerability reduction, rather than through explicit adaptation policies (Schipper 2007:3). Policy mediates both knowledge production and distribution: asset distribution can be regulated, protection to non-state actors can be granted and network infrastructure can be provided. Ideally, it is a two-way route, where communities’ voices will be heard in policy formation (Ensor & Berger 2009:28).

A lack of access to resources and very little say in policies that control these resources cause poor communities to struggle with mediating climate change (Ensor & Berger 2009:2). Mediation is, however, also influenced by the perception of opportunities and impact, which are influenced by information management, capacity to learn and motivated stakeholders (Ensor & Berger 2009:24; Wolf 2011:11). Building adaptive capacity should ideally lead to innovation in responding to and shaping changes (Ensor & Berger 2009:26).

Human interventions have increasing influence on nature’s impact on human society which leads to a denser integration of human and natural systems (Hernes 2012:156). External boundaries of the human system need to be reset in a sustainable and holistic manner, broadening the focus to more than immediate quandaries, targeting ‘policies, institutions and attitudes that establish enabling conditions’ before turning to technology and infrastructure (Schipper 2007:6).

## Perceptions of climate and beliefs

Keeping this idea of fairness and justice in mind when intervening from the outside, the inner workings of autonomous adaptation as specifically influenced by belief systems should be studied to contextualise policy. The above background and conceptualisation indicate that adaptation can be hampered by belief systems but does not make clear exactly how this happens. An empirical study of how climate change challenges beliefs, and in turn how belief systems challenge climate change adaptation, gives insight into this process.

Beliefs about the self, the climate and humankind’s relationship to the environment can be studied by means of Q-methodology, as it is particularly suited for studying subjectivity (Meier 2004). It is a Gestalt procedure that indicates a social construction of sorts, where the focus falls on a specific arrangement of themes (and in this case beliefs) favoured by a group of participants (Watts & Stenner 2005:70–71).

Perceptions of participants from three rural communities in the North-West province were sampled. This province is an important agricultural region but plagued by droughts. Thirty participants from each site were randomly selected and contacted with the help of a community gatekeeper. These communities are poor, with most participants living in housing structures in informal settlements that do not provide adequate protection against the natural elements. On top of that, informal settlements are often built in exposed areas (Griffin 2012).

The Government of South Africa considers climate change as ‘one of the greatest threats to sustainable development’ and the effects of climate change are disproportionately felt by the poor (Department of Environmental Affairs [DEA] 2011:9). Population growth, being situated in an increasingly water scarce area, social and economic problems and continuously changing water management priorities and structures, all add pressure to an already dire situation (Dennis & Dennis 2012:417).

Qualitative data were captured through semi-structured interviews consisting of two basic questions: ‘What do you think about the climate?’ and ‘Do you think your beliefs about the climate can change?’ Q-methodology was then used as a quantitative tool to analyse these data. From the concourse, 40 statements were identified from the interviews for use as the Q-sample. These statements had to be not only ‘broadly representative’ (Watts & Stenner 2005:75) but also condensed in format. The concourse is a representative collection of statements covering all possible opinions about the chosen subject that the respondents might have (Van Exel & De Graaf 2005:4). Brown (1993) describes it as ‘the flow of communicability surrounding any topic’.

In phase two, 15 of the original respondents were randomly selected to do a Q-sort according to a free distribution grid. The grid was arranged in a Likert scale with seven options, ranging from -3 (strongly disagree) to +3 (strongly agree), with 0 (zero) as neutral. For the Q-sorting in phase three, less participants (this time seven or eight per site) were used. They had to rank each item ‘in a fixed quasi-normal distribution’ and ‘along a simple, face-valid dimension’ (Watts & Stenner 2005:77). As Van Exel and De Graaf (2005:7) recommend, an interview was conducted after each Q-sort.

When it comes to the interpretation, there are different perspectives on the data set (Watts & Stenner 2005:81). Things like setting up the final Q-set and interpretation of the factors can almost be seen as an art, but the analysis of data is technical and objective. It consists of the following five stages: creating the correlation matrix, factor analysis, determining factor loadings for each Q-sort, factor rotation and finally the calculation of factor scores and difference scores (Van Exel & De Graaf 2005:8–9). The factor score leads to a composite or idealised Q-sort for each factor. The Difference scores leads to the identification of distinguishing statements. The factors, which are interpreted into summarising accounts, represent operant clusters of subjectivity (Van Exel & De Graaf 2005:1) and in this way people, not tests, are correlated.

No single story exists, therefore inconsistency and complexity should not surprise (Previte, Pini & Haslam-McKenzie 2007:142). The benefits of Q-methodology according to Billard (1999:365) are its use of reflexivity, raising consciousness, empowering the participants and the creation of ‘locally situated understandings’. These effects were observed and strengthened by hosting a small workshop on climate change after the phase three of the project which involved the Q-sorting interviews.

## Results of the Q-sort

### The five factors

The five factors (see below) that emerged from the Q-sort explain 58% of the sample’s variance. The first factor is the most prevalent, with a 20% contribution, followed by 12% from factor 2, 10% from both factors 3 and 4, and 6% from factor 5. Four defining sorts were flagged for factor 1 and 2 each, two for factor 3 and 4 and one for factor 5. Sorts of factor 1 and 4 have the closest correlation in terms of factor scores. All the sorts fit multilaterally and indicate that no participants fit purely into any one factor.

Here follows a summary of each factor, using significant statements from the sorts to create a short narrative. Each factor was named according to the unifying theme that explains the factor’s array best as per the author’s interpretation.

#### Factor 1: Naturalist collectivist

Climate change is nature’s way of reshaping itself. It’s part of our daily life and influences us emotionally as well as our environment via the growth of crops and production of food. The problems caused by climate change should be addressed now to prevent future disaster. We can do it by coming together and discussing it. I feel strongly that climate change is not a punishment for our sins, nor is it caused by traditional healers or the fighting of the ancestors. Educating people will not cause bad luck or anger the ancestors.

#### Factor 2: Religious (contradictory in terms of human agency)

God is in control of everything and he created the climate (it is not affected by traditional healers or fighting of the ancestors). There’s nothing wrong with the climate, it is natural and unpredictable. Our behaviour has no influence on it, but we have to respect the environment. It was better in the older days and returning to those ways (living closer to nature and others) might improve the situation. At the same time, I do believe technology can have an influence. I also strongly believe that young people can teach older people about the climate. I am open to change my beliefs and learn more.

#### Factor 3: Religious determinist

God is in control of the climate, but we as humans have a big influence on it: fossil fuels and pollution are related to climate change. It is a problem, because climate influences our crops and food production – it plays an important role in our lives. The climate was better when I was younger and the next generation will have it even worse than we do today if we don’t do something about it now – it is not the government’s responsibility. To change beliefs is difficult. I am convinced of my beliefs and the only way that I will change it is if I see proof to the contrary. Climate change may be a sign that the world is ending but maybe we can fight it with technology.

#### Factor 4: Activist collectivist (technology/human)

The climate is definitely changing and it is because of the burning of fossil fuels and pollution, we ought to switch to sustainable technologies. Everybody has the right to know about these issues. We should stand together and unite. It is people who just want to make money that harms the environment. Climate change is not a sign that the world is ending and population growth does not affect it.

#### Factor 5: Structural (contradictory in terms of time)

I’m open to change my beliefs; we learn new things all the time. We have to find solutions to climate change, because the climate plays an important role in our lives: laws to protect the environment should be drafted and the government must give people information about climate change. The next generation won’t be influenced by our behaviour today. It will not help to return to the ways of the past, young people can rather help older people to catch up on knowledge regarding climate change. Because of their beliefs, it might be difficult to educate some people. The climate is complicated and unpredictable. It’s possible that traditional healers can cause climate change.

In addition, looking at selected statements in isolation can shed further light on the functioning of belief systems in this context. The statements that specifically relate to the participants’ perceptions of belief revision have been included in Table 1 and will be discussed below.

**TABLE 1:** Q-sort values for statements about belief revision.

Number	Statement	Factor 1	Factor 2	Factor 3	Factor 4	Factor 5
33	It is difficult to educate people about climate change because of their beliefs.	0	-1	0	-2	2
34	It is possible to change my beliefs when someone else tells me to.	-1	-2	-2	0	-2
35	In order to change our beliefs about the climate, we must sit down and discuss the matter.	2	1	1	1	1
36	My beliefs can change if I see in reality that things are different from what I believe.	2	2	2	0	2
37	My beliefs about the climate can change when I feel less vulnerable.	0	0	-2	0	0
38	I am open to change my beliefs, because I learn new things all the time.	1	2	0	0	3
39	It is not possible to change my beliefs.	-1	-1	1	0	-2
40	The climate influences how people feel emotionally and that may cause changes in their beliefs.	1	0	-1	-1	0

These statements don’t offer polarising views, with the exception of statement 38 for factor 5. A reason for the lack of polarising viewpoints might be that personal beliefs are not often considered consciously and therefore are not available for critical analysis. For this reason, it is somewhat abstracted and it is practically not engaged with. In communities that traditionally have a more concrete worldview, this might mean that abstractions are not prioritised. Four out of the eight statements about the participants’ views about beliefs rank in the eight most agreed upon statements, which indicate that people tend to hold similar neutral views about their way of believing.

Another way to study beliefs is to look at concrete examples thereof. By selectively studying statements concerning the cause of climate change, time orientation, solutions to climate change (also indicative of beliefs about human agency and opportunities) and meta-beliefs (the participants’ perceptions of beliefs), specific patterns of belief can be observed. The first factor strongly defines what climate change is not causally attributed to: traditional healers, fighting of the ancestors and punishment for people’s sins, although it shies away from pinpointing what the cause might be. Participants whose sorts load high on this factor seem to be fairly conscious of the whole spectrum of time (past, present and future) but emphasise the now in terms of action. They feel strongly that solutions need to involve the community as a collective, and this collectivism is also manifested in their statements on beliefs. It is only as a group, and by seeing that reality is different from what they believe, that they expect their beliefs to change.

Participants from factor 2 ranked factors about cause and beliefs most strongly. God is seen as the primary cause of climate change, with technology playing a secondary role, and they feel that beliefs can be changed through education. Participants seem to be slightly past oriented and perceive that returning to ways of the past will be the best solution for climate change.

According to Q-sorts representing factor 3, God as well as fossil fuels and pollution are the definite causes of climate change. This factor reveals a broad consciousness of time with high rankings in statements implying past, present and future time conceptions. Statements that can be categorised under *possible solutions* and *the impact of human agency* ranked neutral. Factor 3 participants are seemingly closed to changing their beliefs, except if the reality clearly proves them wrong.

The fourth factor displays a strong regard for technology, pollution and fossil fuels as causes of climate change. The participants in this factor feel strongly about collective action in the present and recommend the use of sustainable technology. Their time orientation is focused on the here and now. Rankings on belief statements are prevalently neutral but indicate an awareness that it can be difficult to change. In correspondence with their concrete attitude and collectivist approach, personal belief statements are all ranked at zero, while statements including elements of action (e.g. educate and discuss) and referring to groups of people are more decidedly ranked.

Factor 5 participants see climate change as either natural or possibly caused by traditional healers. They have a future time orientation (although their agreement with the statement ‘the next generation will not be influenced by our current behaviour towards nature’ is contradictive) and feel strongly about finding solutions to climate change problems. Beliefs were ranked decisively, especially in comparison to the rankings made by other factors in this regard. This can be indicative of a deeper structural consciousness, which would correspond with their beliefs about government. The government is seen to be responsible for climate change intervention by drafting laws to protect the environment. These participants are very open to changes in their own beliefs, although they regard others’ beliefs as more set.

## Perceptions of cause and risk

Qualitative analysis from the interviews will add further depth to the Q-sort results. Beliefs regarding cause are linked to perceptions of controllability, which is related to action, and for this reason important for adaptation. For instance, participant^2^ illustrates how a high perception of controllability translates into belief flexibility, when he says:

‘if they say yes, there is a solution for you to go and change the tsunami, I would…I would change what I know about tsunami, I would go and try to change it.’ (01-SS-Ventersdorp)

Participant 01-BJ-Ventersdorp states that the time that his *‘*perceptions towards heat and everything can change’ will be when there are ‘mechanisms in place’ that ‘protect us against the climate change’.

The majority of people linked pollution and industrialisation to climate change but not necessarily in a logical or linear (causal) fashion. For example, respondents would mention the air that they breathe being polluted, leading to sickness, leading to climate change.

Participant 06-KM-Ventersdorp talks about open rubbish dumps in residential areas and how they cause odours which is bad for our health and the climate. For her, it is a sign that people no longer respect each other or the environment. Many participants mention the ozone layer as well, linking global warming to too much sun that enters. Overall, it seems that climate change is part of a bigger, more inclusive concept pertaining to general well-being.

Sometimes, however, climate change is comprehended more rationally, with an appreciation of the wider consequences. For example, participant 03-SS-Ventersdorp refers to droughts influencing farmers, which in turn leads to higher market prices for food. Taking into account that more severe droughts are the biggest visible effect of climate change in the area, surprisingly few participants mention it. Examples given to illustrate climate change include more erratic patterns of rainfall and temperature, and earlier seasonal changes. Unpredictability is seen as recent, especially by older people who often related how they used to be able to predict certain weather events but cannot do so anymore.

Naïve understandings sometimes involve fear. As participant mentions:

‘… if we cut out traditional rituals, we will be hit by floods, we will be hit by a huge climate change because now everything no longer happens normally, we have already changed it.’ (03-RM-Ikageng)

Beliefs regarding causality can be a window into underlying values (cf. the structure and function of belief systems) and therefore should be expected to be difficult to change. As categories for solution and attitudes are determined by this, a premium placed on tradition and traditional beliefs can cause a resistance to unfamiliar adaptive measures.

Climate change as hazard is perceived in different ways. Feelings of helplessness are illustrated by the following quote:

‘Instead of adapting they challenge the environment and … Technically, they challenge those who know… So, they resist adaptation but somewhere, somehow, they are forced to adapt because there is nothing that they can do… Cannot afford those… Air conditioners or those air cons… Although mentally they resist adaptation but… they just adapt. But some adapt, some challenges, some they just … They don’t know what to do.’ (04-AB-Jouberton)

Some participants are neutral or even positive about climate change, for example, participant 04-KM-Jouberton mentions that winter is now more tolerable, but change is seen as a threat by others. Participant 04-GP-Ventersdorp mentions how clouds have changed: *‘*The clouds are scary… Sometimes He (God) just scares us and we just have to accept (it).’

Such emotional responses may be the result of a disturbance in the belief system following unsettling climate events. This may lead to additional feelings of insecurity (Hernes 2012:96–116) and cause defensive action in the form of resistance to belief revision. Affect also influences risk perception. Wyer and Albarracín (2005:305) connect the anxiety one feels about an event to the approximation of the likelihood of that event. Risk perception influences a community’s motivation to change, which can include a change in beliefs (Wyer & Albarracín 2005:305).

### Belief processes

Quote by a participant:

‘I have a strong belief because the things that we see, that has been happening at all of our times but up until a recent years it seems as if there are changes most of the time the clouds are showing the signs of the rain but when we are expecting the rain it does not rain, so we take that it is the change of life that people and the process of our life at the present moment, in the past there was no killings, wars, things like that right now bloodshed and killings it seems as if have brought a huge change in the process of our cause.’ (05-RM-Ventersdorp)

This quote illustrates how two beliefs can be linked. The participant observed gradual changes in the weather that she could not explain, while during the same time moral decay was witnessed. Both of these observations likely lead to the same feeling of things not quite being right, and were therefore linked in a causal way. This will be possible only when the explanation is consistent with the central structuring component of the belief system. It is one possible way in which beliefs are formed.

Established beliefs narrow the possible explanations of a phenomenon. Participant 02-SS-Jouberton talks about older people with strong traditional beliefs and how ‘if something is done about the climate change, they will believe that the ancestors are happy and they will continue pleasing them’. When the opposite happens and climate change gets worse they will still believe that the ancestors are responsible – this time being angry because they have not pleased them.

A belief is more established when it is integrated into a belief system, and will consequently resist change, as the following quotation illustrates:

‘I believe, I know like most of the time in studies … I read and I take something inside in my own way. So, I won’t change my beliefs because it’s something that I know. Something that I’ve learning when I grow up’. (01-AB-Ventersdorp)

Naïve scientific explanations of climate change can be held next to more traditional views. Participant 04-RM-Ventersdorp explains climate change as caused by people in factories which cause a lot of ‘smokes’ that then ‘leads to increased heat’ but also by ‘that something that stays in the river, that I will not be able to say it by name’. Beliefs that are seen as contradictory are either changed or arranged in a hierarchy. Participant 01-RM-Ventersdorp used to believe that climate was natural and controlled by God but says that he changed these beliefs because he now believes climate is controllable. Participant 03-KM-Jouberton says that she does not ‘undermine anything’ and ‘do believe that … smoke pollutes air’, but she ‘believe(s) mostly in God’.

Cultural identity forms a barrier in terms of authority beliefs (cf. the structure and function of belief systems), as ‘the white people’ don’t understand culture (‘westernised people do not have cultures or traditions’ according to 05-SS-Jouberton) and therefore could not possibly *know* in the same way. Grasping this concept intuitively, participant 02-SS-Jouberton suggests that the only way that some people’s beliefs about climate might change is if your trick them: ‘…call a sangoma (traditional healer) … and convince them to convince the older people and tell them the ancestors say it.’ Also see below quotes:

‘As African, yeah, we have rituals … you have to burn wood for cooking or doing whatever you have to do … so there’s a lot of smoke. When comes to the fact that you have to tell a person that you know what you’re doing is not good for the environment so what and so forth, that person will not be convinced by what you’re telling them … they’ll raise the fact that, no you seem like to be forgetting that I’m an African. I’m not westernised or something.’ (05-SS-Jouberton)

‘The black community, because our culture will teach us another thing and then when we see things in television information, it will be something like ‘Nah, this is a white man’s perspection (sic)’ … it challenges … That why when you come to us, you tell us about climate change, it won’t be easy for us to believe you. Because based on how we grew and how we were taught … that, ah, ah, ah … this is the cultural way. Because of, we see nature differently, as (than) the white man.’ (04-AB-Jouberton)

The quotes above further illustrate how culture can be a very real barrier to change. In a similar vein, the issue of knowledge as power was touched upon. Participant 04-AB-Jouberton shares his belief that religious beliefs were used during the colonisation of South Africa to pacify the indigenous people, while robbing them of precious raw materials. That is to say that when you change someone’s core beliefs, it becomes easier to influence them.

Specialist knowledge can create the illusion of control. Participant 01-RM-Ventersdorp deduced that meteorologists control the weather because they know what the weather will be like and therefore ‘it’s no longer … natural’.

Climate change and climate change education is a necessary process that holds its own challenges. The following quotation illustrates how the structure of a belief system can resist information based on presentation:

‘But not to convince; because if you try to convince man, sometimes people become very personal and people will think not that you are taking them for granted. And you think that you are the alpha and omega about whatever you are trying to let their minds be changed.’ (04-SS-Ventersdorp)

As was pointed out earlier (cf. the structure and function of belief systems), consistency with self-esteem receives priority over other types of consistency in a belief system. If identity is challenged, the belief system will act in irrational ways to protect it. In this regard, Adger et al. (2009) urge that local knowledge should be acknowledged and cultural identity should be safeguarded in planned adaptation. If identity is challenged, an emotional response is elicited and more coherent relationships between new and existing beliefs will be necessary for belief change (Thagard & Findlay 2010).

There is a real willingness to learn amongst many of the participants, who see a change in outlook as a prerequisite to adaptation. Participant 01-SS-Ventersdorp notes the importance of flexibility of belief, recommending that ‘we must always believe that – there’s what we call probability in life… you must always be prepared for disappointment’. He stresses the communal effort needed, advising that ‘we must – as people … change our mind-set … we must now starting to adapt or put our mind-set in the very same place’. Some participants mentioned schooling and even Internet-based information, but a specific need for accessible and relevant information in the community was expressed more than once.

### Factor-specific challenges and opportunities

Participants from each factor will experience different challenges because of their different worldview narratives. Factors 1 and 4 might experience culture and social fragmentation to be more of a barrier because of their emphasis on collectivism. Both of these factors place emphasis on seeking a solution, without attaching explicit importance to beliefs. In contrast to factors 1 and 4, factor 5 also emphasises solutions and human agency but does so while simultaneously acknowledging the importance of beliefs and systems, which suggests a bigger picture view. Factor 3 displays the biggest resistance to belief change, which is probably a result of religious determinism. Factor 2 seems more optimistic and open to change. Factors 2 and 4’s belief systems are possibly less integrated because of contradictory views (in terms of human agency and time perception respectively as indicated in their narratives). By using this instability in cohesion as leverage, beliefs could be changed.

Opportunities form an integral part of adaptation and can be customised according to each factor’s beliefs. Climate change can be utilised as an opportunity to gather the community to unite around a cause for factor 1. For factor 2 learning through technology and for factor 3 learning about technology might be a prospect. The profile of factor 4 lends itself to building a large network influencing more human ways of life and a socially responsible economy. Factor 5 participants will be able to collaborate with government.

## Conclusion

Adaptation should ideally be guided by governing bodies externally in support of adaptation processes within a community. According to the National Climate Change Response White Paper, South Africa faces ‘future drying trends and weather variability with cycles of droughts and sudden excessive rains’ (DEA 2011:8). However, in advising policy, one should keep in mind the distinct views, even within one community, and consider how one can address each effectively. The systems and structures that will advance the unity of the community should be prioritised and perhaps contested issues should be sidestepped. Eriksen et al. (2011) advise that vulnerability in its wider context should be kept in mind, as well as the way different values will affect adaptation outcomes.

Elements shown to increase resistance to belief revision are a high integration of established beliefs concerning climate change, social embedment of beliefs, a high importance attached to certain beliefs, a narrowed perception (because of cultural beliefs), perceived frightening consequences, negative referents stating a contrary belief and threat to ego or identity. Properties that facilitate belief change are perception of controllability, clear evidence and a willingness to learn.

On a more global level, when it comes to the issues surrounding climate change, our way of thinking needs to change and the start of that process lies in mapping where we currently are. This study has attempted to create such a map. It explored possible challenges created by the interrelationship between climate change and belief systems in order to make better decisions in terms of adaptation.

## Footnotes

1 Kuhn discusses beliefs in terms of paradigms. In the sense referred to here a paradigm can be defined as a communal belief system with shared obligations to values and other symbolic generalisations.

2 The qualitative data from the interviews were coded according to the number of the interview, the interviewer’s initials and the name of the community.
